# Children and adolescents presenting to chiropractors in Norway: National Health Insurance data and a detailed survey

**DOI:** 10.1186/s12998-016-0107-x

**Published:** 2016-08-01

**Authors:** Anna Allen-Unhammer, Francis J. H. Wilson, Lise Hestbaek

**Affiliations:** 1 Markveien Fysikalske Institutt, Markveien 25, 0554 Oslo, Norway; 2 Anglo-European College of Chiropractic, Bournemouth, Dorset BH5 2DF UK; 3 Nordic Institute of Chiropractic and Clinical Biomechanics and Institute of Sports Science and Biomechanics, University of Southern Denmark, Campusvej 55, Odense M, DK-5230 Denmark

## Abstract

**Background:**

The idea of chiropractors treating children is controversial, yet many parents seek chiropractic care for their children. The reasons for this are not well documented. Part one of this study aimed to document the profile of all paediatric patients consulting Norwegian chiropractors in 2013 using National Health Insurance data (NHI) with regards to age, gender and primary complaints. Part two aimed to provide a more detailed description of these patients in the form of a descriptive, paper-based survey.

**Methods:**

Part one involved Norwegian NHI data from 2013. Part two consisted of a year-long, paper-based survey. Chiropractors registered with the Norwegian chiropractic association (NKF) were invited to participate via email. Participating chiropractors were assigned one random month to collect data. All paediatric patients (or their parents) during that were asked to complete questionnaires containing information on presenting complaint, consequences of this complaint, age, and mode of referral.

**Results:**

In general, there was good concordance between part one and two of the study in terms of age, gender and presenting complaint. The youngest children constituted the largest paediatric group in Norwegian chiropractic practice. Musculoskeletal problems were the most common reason for children visiting a chiropractor in all of the age categories, according to NHI data. Part two of the study found that one-third of young school children and adolescents reported pain lasting longer than 1-year. Eleven percent of children found that their complaint had severely affected their mood and 22 % felt their activities were very limited by their condition. Referrals from healthcare professionals were uncommon. The survey’s results were based on 161 completed questionnaires, received from 15 % of the Norwegian chiropractors.

**Conclusions:**

Musculoskeletal problems constituted the most common presenting complaint for children and adolescents presenting to Norwegian chiropractors. A sizable proportion of children seemed to be adversely affected by their complaints in terms of low mood and limitations of daily activities. Chronic presentations were not uncommon. The small sample size of the survey demands that the results be interpreted with caution.

## Background

Every year, around 7 to 8 % of the Norwegian population consults a chiropractor, according to the Statistics Norway/Census (Statistisk Sentralbyrå’s) health survey [1]. The number of parents seeking chiropractic care for their children has increased substantially in recent years [2, 3].

Yet, in the midst of this increasing trend, whether or not children should receive chiropractic care remains a contentious issue. A particular concern has been the wide range of paediatric conditions that are claimed by some chiropractors to be amenable to chiropractic treatment. While pain conditions remain the most common reason for adults seeking chiropractic and other forms of CAM, this is less clear in children [4]. Children have been reported to present for a variety of reasons that can be grouped into two main categories: musculoskeletal (MSK) conditions, for example torticollis, neck pain and lower back pain, and non-musculoskeletal conditions (non-MSK), such as enuresis, infantile colic (now more commonly known as excessive crying) and asthma [5–9]. The need for documentation of descriptive characteristics in paediatric patients presenting to chiropractors is warranted.

The majority of the studies documenting the descriptive characteristics of this patient group in chiropractic practice have come from English-speaking countries, but descriptive surveys have also been published in Sweden [10], Holland [11], and Denmark [12–14].

One previous study reported on the basic features of the Danish chiropractic patient population using questionnaires distributed to chiropractic clinics in 1999 [12]. A relatively large proportion of infants (1.5 %) were reported to have presented to chiropractors in Denmark. A nationwide study on children under 18 years of age in Denmark revealed similar findings [14], showing that babies accounted for the majority of paediatric patients (40 %) that presented to Danish chiropractors.

A Danish study on CAM conducted in 2003 at Odense Hospital on 622 participants found that chiropractic patients were typically less than 1-year old and had sought such care to address joint problems and/or gastrointestinal symptoms [15]. Hestbæk *et al’s* study [14] agreed with those results. Excessive crying, infantile colic and stomach problems were found to be the most common presenting complaints among the babies. However, musculoskeletal problems were most prevalent (26 % in preschool children versus 75 % of adolescents), followed by headaches (13 %) in school children and adolescents. Presentation due to non-MSK complaints such as asthma, allergy and otitis media were most frequently found in pre-school children. This was also concordant with a survey conducted on 749 children, between the ages of 4 and 18 years of age in three German schools [16]. This survey found that 61 % suffered from headaches, 43 % had abdominal pain, 34 % had limb pain and 30 % had back pain. However, the proportion of each age group suffering from these complaints was unclear from the article.

There is growing evidence to support the apparent chronicity of complaints that afflict children. Hartvigsen et al. found that 21 % of Danish patients (including adults) reported pain lasting for more than 1-year [12]. This finding was later reinforced by Hestbæk et al., where approximately one-third of children were reported to have had symptoms for more than 1 year before seeking chiropractic care and almost one-quarter of patients with musculoskeletal complaints reported symptoms lasting for more than 1-year [14].

Up to 50 % of children and adolescents are estimated to experience back pain or other musculoskeletal problems in a 1-year period and around one-third of these will have recurrent pain episodes [17–19]. A 3-year prospective study found that 58 % of Norwegian adolescents (mean age of 15 years) reported back pain during the previous year, while 32 % reported back pain lasting for more than 7 days during the previous year [20]. There is evidence to suggest that the paediatric population suffering from back pain is more likely to have recurrent pain in adulthood and that musculoskeletal problems that begin in early life may track into adulthood, similar to the pattern seen with cardiovascular disease [14, 21–23]. According to the Global Burden of Disease Study, back pain remains the leading cause of years lived with disability in Norway (Institute for health Metrics and Evaluation, 2013). It also remains the main cause of work absenteeism and disability benefits in Norway [24].

While some parts of the world have made some progress in documenting demographic features of chiropractic patients less than 18 years of age, data in Norway is lacking. Part one of this two-part study aims to document the profile of all paediatric patients consulting Norwegian chiropractors in 2013 using National Health Insurance data (NHI) with regards to age, gender and primary complaints. Part two aims to provide a more detailed overview of these patients in the form of a descriptive, paper-based survey in terms of i) primary complaints in babies versus toddlers (since NHI data only report from 0 to 4 years of age combined); ii) duration of primary complaint, consequences of these complaints, mode of referral; and finally iii) the socioeconomic background of the care-seeking families.

## Methods

### Part one: Norwegian NHI data

Norwegian National Health Insurance (NHI) data from 2013 were sought, documenting descriptive data on all paediatric patients presenting to Norwegian chiropractors with regards to age, gender and diagnosis. The Norwegian registry (Helfo), which provided this data, accumulated the number of treatments administered by chiropractors according to diagnostic codes given. When a patient consults a chiropractor and a diagnosis is registered in the patient’s file, this information is then relayed to Helfo. Helfo is an external agency under the Directorate of Health in Norway, which provides partial reimbursement for chiropractic treatment. The chiropractor is reimbursed directly with regards to the coverage the patient is entitled to, under the Norwegian National Health Insurance Scheme. This is so that the patient avoids having to apply for Helfo reimbursement themselves. There is no statutory requirement that chiropractors must be registered with Helfo, but Helfo stated (on questioning), that to the best of their knowledge, all chiropractors who were actively practising in Norway had signed an agreement with Helfo. Since all diagnoses from chiropractic consultations were registered with Helfo, this provided an accurate account of reasons for paediatric patients consulting Norwegian chiropractors.

Relevant data were retrieved and summarised in Microsoft Excel.

### Part two: Survey results

Part two of the study was a paper-based survey. The methods, questionnaires and data collection procedure used in this study were based on those utilised by Hestbaek et al. [14]. Permission was granted from these authors to use their questionnaires. For the current study, the questionnaires were translated from Danish to Norwegian by a Norwegian chiropractor and then translated back into Danish by two independent persons in order to check accuracy, with regards to wording of the questions.

Discrepancies exist regarding the way in which different age groups of children are defined in the research literature [25], which can make comparisons between studies difficult. Attempts have been made to standardise age groups among children. For example, age stages were defined in 2011, according to *Eunice Kennedy Skriver* National Institute of Child Health and Human development (NICHD) terminology in the United States. These age stages have since been recommended for establishing consistent age groups in randomised controlled trials [25]. Yet, there seems to be considerable overlap between different age groups, depending on which institution one refers to. Table 1 serves to illustrate the varying definitions of children under the age of 18.

**Table 1:** Illustrates the variable definitions used and overlap of different age groups of children

Name	Definition
Preterm neonate	Period at birth when a newborn is born before full gestational period.
Term Neonate	Birth to 27 days. Newborn up to 1 month. A newborn infant, or neonate, is a child under 28 days of age.
Infant/Infancy	28 days to 1 year of age. 1 month to 2 years. 0 to 1 year of age.
Toddler	13 months to 2 years. 1 to 3 years.
Preschool child	3 to 5 years.
Early childhood	2 to 5 years.
Middle childhood	6 to 11 years.
Child	2 to 12 years.
Young teen	12 to 14 years.
Teenager	15 to 17 years.
Adolescent	12 to 16 years. 10 to 19 years.
Early adolescence	12 to 18 years.
Late adolescence	19 to 21 years.

https://www.nichd.nih.gov/health/clinicalresearch/clinical-researchers/terminology/Pages/current.aspx

^a^ *NICHD Eunice Kennedy Shriver* National Institute of Child Health and Human Development

^b^ *FDA* US Food and Drug Administration

^c^ *WHO* World Health Organisation

^d^ *CDC* Centers for Disease Control and Prevention

Three questionnaires were used in order to collect descriptive data from different age groups of children for part two of the study. Part two of the study was based on that of Hestbaek et al. (2009) with regards to questionnaires and methodology and therefore adopted the same divisions between different age groups:

- Infants: 0–1 year of age.
- Pre-school children: 2–5 years of age.
- School children and adolescents: 6–11 years of age and 12–17 years of age respectively.

Within the above framework, the term “baby” referred to 0–3 month olds while the term “toddler” referred to 13–23 month olds.

The categories “school children” and “adolescents” used the same questionnaire. The only modification in relation to the Danish study [14] was the addition of three questions on parental education and occupation as a reflection of socioeconomic status (SES).

The procedures and questionnaires were pilot-tested in two chiropractic clinics on a total of 20 patients between April and June 2012. As a result, the wording of the questions changed slightly to enhance understanding.

### Variables

The questionnaires contained information on age, gender, postcode, primary complaint, duration of primary complaint, parental education and occupation (SES), mode of referral, limitations of activities of daily living (ADLs) and whether mood was affected. The latter two were only included in the questionnaires for school children and adolescents.

### Study procedure

Data collection took place between December 2012 and November 2013 to account for seasonal variations [14]. The month of July 2013 was omitted due to school summer holidays. All chiropractors in Norway, registered with the Norwegian chiropractic association (NKF), were invited to participate via individualised emails. Each chiropractor who agreed to participate was assigned 1 month in which questionnaire data was collected and were sent six questionnaires. The questionnaires, instructions to the chiropractor, patient information sheets and return envelopes were sent to all of the participating chiropractors 2 weeks before their allocated data collection month. The questionnaire was filled out by the patient or parent prior to the consultation to ensure that the answers were not influenced by the chiropractor. The child or parent was asked to complete the questionnaire if the child was 16 or under, whereas the adolescent was asked to complete the questionnaire if they were 17 or 18 years of age.

The forms were collected by the chiropractors, sealed in an envelope and returned anonymously to the project leader (AAU) by post. If the patient or the patient’s parent or guardian did not want the child to be involved in the study then a blank questionnaire was returned. Participating chiropractors received a reminder by email in the first week of their data collection month to ensure that they had received their questionnaires and had started to collect data.

### Data analysis

Response data were manually entered into an Excel spreadsheet and analysed using this program. Double entry of data from a 10 % sample revealed an error rate of less than 1 %, which was considered to be satisfactory. Descriptive statistics were used to analyse single variables and results were descriptively reported in tables and figures.

## Results

### Part 1: Norwegian NHI data

This data was based on 28 603 chiropractic patients under the age of 18 that received a total of 114 258 treatments.

### Gender and age

Overall, the gender distribution was almost equal between boys and girls between the ages of 0–14 years, see Table 2. When considering the individual age groups, there was a slight predominance of boys, except in the 15–17 year age group where there were more girls (57 %). Children between the ages of 0–4 years were the most common paediatric patients in Norway. This age group was not further subdivided and thus data on the number of babies and toddlers were not available.

**Table 2:** Gender distribution according to Helfo data (2013)

Gender/Age of child	0–4 years	5–9 years	10–14 years	15–17 years	Total
Female	4750 (45.8 %)	1599 (43.4 %)	3681 (49.5 %)	4764 (57.0 %)	14,794 (49.4 %)
Male	5610 (54.0 %)	2077 (56.4 %)	3742 (50.4 %)	3658 (42.4 %)	15,087 (50.4 %)
Unknown	20 (0.2 %)	5 (0.1 %)	8 (0.1 %)	9 (0.1 %)	42 (0.1 %)
Total	10,380 (100 %)	3681 (100 %)	7431 (100 %)	8431 (100 %)	29,923 (100 %)

Helfo: is the Norwegian registry that provided the National Health Insurance data on the accumulated the number of treatments administered by chiropractors, according to diagnostic codes given

### Primary complaints below 4 years of age

(i) Primary complaint

Musculoskeletal problems accounted for almost two-thirds of complaints (68 %) in children between 0 and 4 years of age (see Table 3), followed by excessive crying/infant colic (14 %).

**Table 3:** Primary complaint according to Helfo data (2013)

Complaint/age of child	0–4 years	5–9 years	10–14 years	15–17 years	Total
Excessive crying/infant colic	1427 (13.75 %)	11 (0.30 %)	1 (0.01 %)	-	1439 (4.80 %)
Disturbed sleep	37 (0.36 %)	2 (0.05 %)	0	2 (0.02 %)	41 (0.14 %)
Stomach	1870 (18.0 %)	40 (1.09 %)	15 (0.20 %)	7 (0.08 %)	1932 (6.46 %)
Ears	20 (0.20 %)	18 (0.49 %)	14 (0.19 %)	5 (0.06 %)	57 (0.19 %)
Nose/throat	0	0	0	1 (0.01 %)	1 (0.003 %)
Musculoskeletal system	6539 (63 %)	2921 (79.35 %)	6917 (93.08 %)	8020 (95.13 %)	24,397 (81.53 %)
Headache	4 (0.04 %)	152 (4.13 %)	95 (1.28 %)	190 (2.25 %)	441 (1.47 %)
Asthma/allergy	4 (0.04 %)	2 (0.05 %)	0	4 (0.05 %)	10 (0.03 %)
Dizziness/lethargy	3 (0.03 %)	6 (0.16 %)	22 (0.30 %)	20 (0.24 %)	51 (0.17 %)
Concentration/hyperactivity	11 (0.12 %)	13 (0.35 %)	3 (0.04 %)	0	27 (0.09 %)
Prophylactic examination	174 (1.68 %)	21 (0.57 %)	11 (0.15 %)	8 (0.09 %)	214 (0.72 %)
Enuresis	30 (0.29 %)	210 (5.70 %)	44 (0.59 %)	3 (0.04 %)	287 (0.96 %)
Other	261 (2.51 %)	285 (7.74 %)	309 (4.16 %)	171 (2.03 %)	1026 (3.43 %)
Total	10,380 (100 %)	3681 (100 %)	7431 (100 %)	8431 (100 %)	29,923 (100 %)

(− = not applicable, 0 = no children presenting with a particular complaint)

Helfo: is the Norwegian registry that provided the National Health Insurance data on the accumulated the number of treatments administered by chiropractors, according to diagnostic codes given

### Complaints: Five to 17 years of age

(i) Primary complaint

Of all the children presenting to Norwegian chiropractors in 2013, almost 82 % of complaints were musculoskeletal in nature (all age groups, including 0–4 years). These increased with age, from 80 % in school children versus 95 % in adolescents.

The presentation of school children and adolescents appeared relatively alike. In contrast, children between the ages of 5–9 years of age differed in that they presented more frequently with non-musculoskeletal complaints. Prophylactic examinations were quite uncommon, and most frequently seen in the youngest age category.

### Part 2: Survey results

Eighty-seven of a possible 598 NKF chiropractors, who had previously agreed to collect data from their paediatric patients, participated in part two of the study, yielding a participation rate of 15 %. Sixty percent of the chiropractors who returned the questionnaires were female. A total of 161 questionnaires were returned. All of the returned questionnaires were completed and filled out properly, thus to our knowledge no children, adolescents or their parents declined to participate in the study. The participating chiropractors collected between one and five questionnaires each, with the majority collecting one questionnaire (62 %).

### Postcodes and data retrieval

There are 19 mainland counties in Norway and data was collected from 14 of these.

### Gender and age

**Fig. 1:**
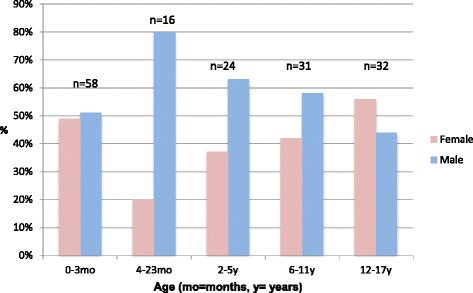
Gender distribution by age of paediatric patients presenting to Norwegian chiropractors 2012–13: *n* = 161, according to part 2 of the study

Overall, there was a slight predominance of male patients (55 %) (see Fig. 1). The disparity between the different genders was most apparent in infants and toddlers category (4 to 23 months), where 80 % of the patients were male, whereas there was a more equal distribution among adolescents (44 % males). Babies (between the ages of 0 and 3 months of age) were the most common paediatric patients in Norway. Of the 74 patients below 1 year of age, 80 % were less than 4 months of age.

### Complaints below 2 years of age

(i) Primary complaint

**Fig. 2:**
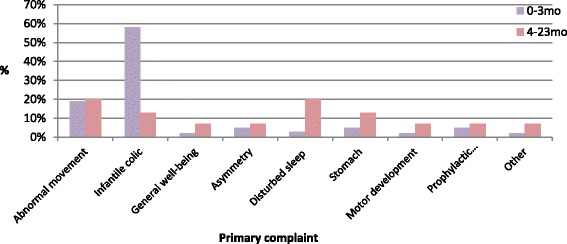
Primary complaints of paediatric patients under the age of two; *n* = 74, according to part 2 of the study

Excessive crying/infant colic accounted for more than half of the visits (58 %) in babies (see Fig. 2). The presenting symptoms in 4–23 month olds (infants/toddlers) were quite variable, consisting primarily of abnormal movement and disturbed sleep (see Fig. 2). Three percent of babies were reported to have musculoskeletal problems that occurred in association with trauma or an accident, compared to 13 % in infants/toddlers.

### Complaints: 2 to 17 years of age

(i.) Primary complaint

**Fig. 3:**
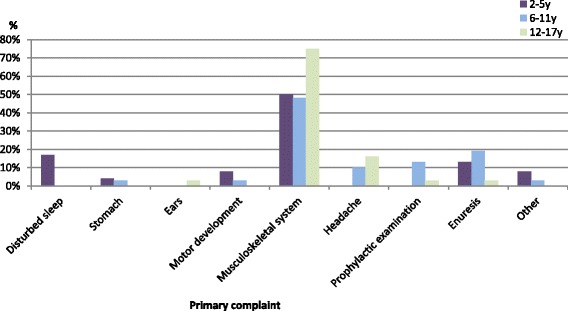
Primary complaints of paediatric patients between 2 and 17 years of age; *n* = 87, according to part 2 of the study

Children between 2 and 17 years of age presented predominantly with musculoskeletal complaints (32 %) (see Fig. 3). These increased with age, from 24 % in pre-school children to 47 % in adolescents. A high proportion reported musculoskeletal problems that had occurred in association with trauma or an accident: 29 % for pre-school children, 19 % for school children and 38 % for adolescents.

The second most frequent primary complaint reported by the 2 to 5 year olds was disturbed sleep (17 %), followed by enuresis (13 %). The school age child’s (6–11 year old age category) second most common primary complaint was enuresis (19 %). Lastly, the adolescent category reported headache to be the second most frequent primary complaint (16 %). Adolescents suffered the most headaches out of all of the age categories. However, this description of secondary complaints is based on small numbers.

The presentation of school children and adolescents appeared relatively alike. In contrast, pre-school children differed in that they presented with more problems relating to the stomach, disturbed sleep and motor development issues. Prophylactic examinations were quite uncommon.

(ii) Consequences: whether mood was affected and whether activities were limited due to complaints.

Eleven percent of children thought their complaint had severely affected their mood and 57 % reported it had had some influence (see Table 4). With regards to activity limitation as a result of complaints: 22 % felt very limited, 51 % felt somewhat limited, while 27 % felt no limitations at all (for age-specific results, see Table 5).

**Table 4:** Consequences: mood affected, according to part 2 of the study

Mood affected/Age of child	0–3 months	4–23 months	2–5 years	6–11 years	12–17 years	Total
Severely affected	-	-	-	3 (9.7 %)	4 (12.5 %)	7 (11.1 %)
Had some influence on mood	-	-	-	15 (48.4 %)	21 (65.6 %)	36 (57.1 %)
No influence at all on mood	-	-	-	13 (41.9 %)	7 (21.9 %)	20 (31.7 %)
Total	-	-	-	31 (100 %)	32 (100 %)	63 (100 %)

**Table 5:** Consequences: activity limitation, according to part 2 of the study

Activity limitation/Age of child	0–3 months	4–23 months	2.5 years	6–11 years	12–17 years	Total
Very limited activity	-	-	-	2 (6.5 %)	12 (37.5 %)	14 (22.2 %)
Some limited activity	-	-	-	20 (64.5 %)	12 (37.5 %)	32 (50.8 %)
No limited activity	-	-	-	9 (29.0 %)	8 (25 %)	17 (27.0 %)
Total	-	-	-	31 (100 %)	32 (100 %)	63 (100 %)

### Duration of primary complaint

**Fig. 4:**
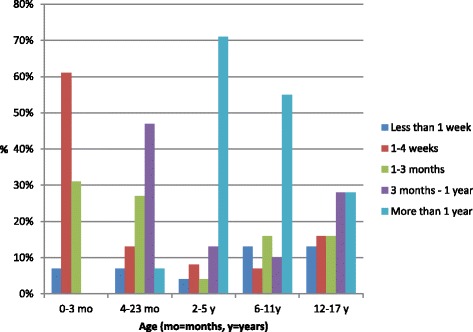
Duration of primary complaint in paediatric patients consulting Norwegian chiropractors 2012–13, *n* = 161, according to part 2 of the study

Sixty-one percent of babies had had their primary complaint for 1 to 4 weeks prior to consulting a chiropractor. The majority of children from 2 to 5 years (71 %) and 6 to 11 years (55 %) had had their complaint for more than 1 year before seeking chiropractic care, as illustrated in Fig. 4. This was also somewhat true of the 12–17 year age group, but to a far lesser extent (28 %) Twenty-two percent of patients with musculoskeletal complaints reported that their symptoms had been present for more than 1 year before consulting a chiropractor.

### Mode of referral

**Fig. 5:**
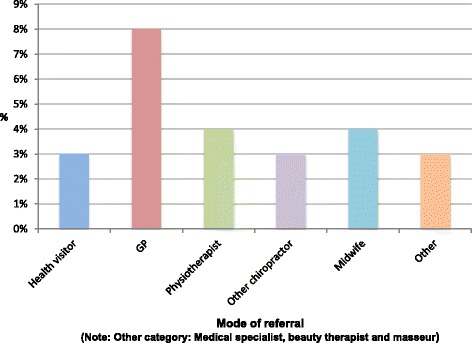
Mode of referral (all age categories combined); *n* = 40, according to part 2 of the study

In total, 25 % of children were referred to chiropractors by people other than family and friends (see Fig. 5). A relatively low percentage of healthcare professionals referred children to Norwegian chiropractors. The largest percentage of referrals came from GPs (16 %), who referred teenagers due to musculoskeletal complaints. Infants/toddlers were referred equally by midwives and physiotherapists (13 %). Health visitors and midwives referred the most babies to chiropractors but the rates were low (7 % each).

### Maternal Socioeconomic Status (SES)

Most of the children’s mothers had a full-time occupation. This finding was most pronounced in the 0 to 3-month old age group (79 %). Sixty-six percent of mothers in all of the children’s age groups had an educational level of university or equivalent. About 4 % of mothers had an educational level of primary school or below.

## Discussion

Part one of this study documented the age, gender and diagnoses of all paediatric patients in Norway in 2013, using National Health Insurance data (NHI). Part two of the study aimed to expand on this, to provide a more detailed description of paediatric patients (from the perspective of the parent/patient), since this data was not available from the Norwegian NHI registry. Only 161 children were included in this survey, which may therefore only be considered a preliminary investigation. The results must be interpreted cautiously because of the small population, but they compared quite well to the NHI data, lending credibility to the survey’s results.

### Comparison with other studies

The finding from both parts of this study that the youngest children constituted the largest paediatric group in chiropractic practice is in line with studies from Denmark and England [3, 14].

Both parts of the study revealed a greater proportion of males than females under 5 years of age. Other studies have reported a similar predominance of males [3, 14]. It has been speculated that this finding may be partly due to boys often being larger at birth than girls, thus musculoskeletal imbalances might ensue [3]. As a result, parents with baby boys may be more inclined to seek chiropractic help. However, one cannot rule out the possibility that other aetiologies were in fact responsible for male babies presenting with excessive crying/distress. A certain degree of professional bias may have occurred, whereby these babies were more likely to receive a musculoskeletal diagnosis/explanation for such symptoms. This is confounded by the fact that the aetiology of excessive crying is poorly understood.

The presenting symptoms of babies were quite variable in part two of the current study, thus reinforcing the findings from previous studies [3, 14, 26]. Excessive crying (previously known as infantile colic) accounted for more than half of the visits in the 0 to 3-month old category. This too, was in line with the findings from several earlier studies [3, 15]. The current study found a clear predominance of musculoskeletal problems among children and adolescents. It should be noted that there was a higher proportion of musculoskeletal complaints reported in part one of the study. This was likely due to it being practitioner-reported data, with possibly more focus on the origin or pain-producing structure (most often musculoskeletal). One must also remain cognisant that chiropractors may be more inclined to give a musculoskeletal diagnosis since they work predominantly with such complaints on a daily-basis. In contrast, data from part two of the study was reported by the patient, where the focus was likely to be on the symptoms (e.g. excessive crying, headache). This predominance of musculoskeletal complaints relates well to the reported increase in prevalence of musculoskeletal problems with age [14, 27, 28].

Presentation due to non-MSK complaints such as asthma, allergy and otitis media were not commonly reported, unlike in previous studies [14, 16]. The reasons for this are unknown. However, the efficacy of chiropractic management in the treatment of non-MSK disorders has yet to be proven [29], and may thus account to some extent for the seemingly low presentation levels of children with non-MSK ailments to chiropractors in Norway.

A considerable proportion of children had been suffering from their complaint(s) (including MSK complaints) for more than a year, which confirmed the findings from previous studies [12, 14, 16]. Furthermore, a study of 564 children purported that about 60 % of children with MSK complaints were still suffering complaints after 4 years [30].

It seems that a substantial proportion of children had been affected adversely by their complaints. These findings are concerning as they could have important implications for both physical activity levels and mental health. Therefore, finding effective treatments to tackle MSK ailments is paramount and as such, further investigation as to whether chiropractic treatment might alleviate such complaints is warranted. This is especially important in light of growing numbers of paediatric patients presenting to chiropractors [31].

Referral rates from healthcare professionals were low, with the exception of a small percentage of GPs who referred adolescents to chiropractors. Two Norwegian studies documented less than optimal communication between chiropractors and GPs [32, 33], which might partly explain the low referral rates in general. Hestbaek et al.*´s* Danish study [14] reported much higher referral rates from healthcare professionals, particularly for babies. This could reflect good inter-professional relationships in Denmark, as a result of chiropractic and medical students being educated within the same institution in southern Denmark.

Positive associations between higher income levels, full-time education after the age of 18, non-manual social class and patients seeking CAM, including chiropractic have been reported [34]. The vast majority of mothers in the current study had a high-level of education, corresponding to having completed a tertiary/university level education. These figures mirror the female Norwegian population in general. More than half of women between 25 and 39 years of age had an educational level of university or equivalent in 2014 Norway’s Census health survey [1], thus representing the most educated age group in the population.

Mothers are thought to be the primary decision-makers when making healthcare decisions regarding their children, including deciding whether to take their child to a chiropractor [35], thus future studies should focus on collecting data from mothers when considering SES as a variable.

### Strengths and limitations of the study

#### Part one

The inclusion of national data from the health register was a major strength of the study. It should be born in mind that a single patient may have been given differing diagnoses during subsequent consultations. Also, a single patient may also have been classified as belonging to one age group during one consultation and then in another age group at a later date, during the course of the same year. Thus the Norwegian NHI data cannot be deemed precise for the individual patient.

#### Part two

Sampling bias was minimised as almost all of the members of the Norwegian chiropractic association (NKF) were invited to participate in the study. However, only 87 chiropractors chose to participate. The majority of whom collected one questionnaire each, which limited generalisability. Differences between participating and non-participating chiropractors were unknown. The results seem to compare quite well to the data from the National register, yet one cannot rule out the possibility of bias affecting the survey’s results. The major limitation of the study was the small sample size. One can only speculate as to the reasons for this low participation rate, as feedback regarding reasons for not being able to participate, were not often given by the NKF chiropractors. Paper-based studies are known to be time-consuming. Since the questionnaires required 10 to 15 min to complete, busier clinics and those with fewer paediatric patients may have been less likely to respond. A study consisting of two surveys found that almost 4/5 of NKF chiropractors reported that less than 10 % of their patients were below 10 years old [32]. Thus, it is possible that chiropractors may not have treated any children during their data collection month. Nevertheless, the data collection period spanned a full year, thereby accounting for any seasonal variations.

## Conclusions

The youngest children constituted the largest paediatric group in Norwegian chiropractic practice, according to both parts of the study. This study highlighted that musculoskeletal problems are common in Norwegian chiropractic patients under the age of 18 and increase with age. The study also revealed a tendency for musculoskeletal complaints to become chronic, indicated by a considerable proportion of patients waiting more than a year before seeking chiropractic care. Additionally, a substantial proportion of children seemed to be adversely affected by their complaints, in terms of mood and limited activities of daily living. In light of these findings, more research should be dedicated to investigate the persistence of musculoskeletal complaints in children and on effective treatment and prevention strategies, with a focus on early intervention in order to prevent chronicity from ensuing. Part one of the study featured a relatively large sample size, whereas part two of the study did not. The small sample size of the latter demands that one interpret the results with a degree of caution.

## Abbreviations

AAU, Anna Allen-Unhammer; ADLs, activities of daily living; CAM, complementary and alternative medicine; GP, general practitioner; HELFO, Helseøkonomiforvaltningen/ Health economics administration; MSK, musculoskeletal; NHI, National Health Insurance; NICHD, National Institute of Child Health and Human development; NKF, Norsk Kiropraktorforening/ Norwegian Chiropractic Association; NSD, Norsk Samfunnsvitenskapelig Datatjeneste/ Norwegian Social Science Data Services; REK, Regional Etisk Komiteen and personvernombudet/ Regional Committees for Medical and Health Research Ethics; SES, socioeconomic status
